# Estrogens and Spermiogenesis: New Insights from Type 1 Cannabinoid Receptor Knockout Mice

**DOI:** 10.1155/2013/501350

**Published:** 2013-11-12

**Authors:** Giovanna Cacciola, Teresa Chioccarelli, Silvia Fasano, Riccardo Pierantoni, Gilda Cobellis

**Affiliations:** Dipartimento di Medicina Sperimentale, Sez. Bottazzi, Seconda Università di Napoli, Via Costantinopoli 16, 80138 Napoli, Italy

## Abstract

Spermatogenesis is a complex mechanism which allows the production of male gametes; it consists of mitotic, meiotic, and differentiation phases. Spermiogenesis is the terminal differentiation process during which haploid round spermatids undergo several biochemical and morphological changes, including extensive remodelling of chromatin and nuclear shape. Spermiogenesis is under control of endocrine, paracrine, and autocrine factors, like gonadotropins and testosterone. More recently, emerging pieces of evidence are suggesting that, among these factors, estrogens may have a role. To date, this is a matter of debate and concern because of the agonistic and antagonistic estrogenic effects that environmental chemicals may have on animal and human with damaging outcome on fertility. In this review, we summarize data which fuel this debate, with a particular attention to our recent results, obtained using type 1 cannabinoid receptor knockout male mice as animal model.

## 1. Introduction

Spermatogenesis occurs in the testis in a stepwise fashion so that committed spermatogonia proliferate and develop into spermatocytes (SPC) to enter meiosis and produce round spermatids (SPT). These undergo a morphological transformation (spermiogenesis) into mature SPT (i.e., spermatozoa), which are differentially released from Sertoli cells (spermiation) depending on the species. In mammals, further transformations occur in the epididymis to form mature spermatozoa (SPZ) suitable for fertilization [1–4]. Spermatogenesis is a process highly conserved throughout vertebrate species and it is mainly under hypothalamic-pituitary control [5–17]. Indeed, it is a hormonally controlled mechanism; apart from gonadotropins and androgens, numerous endocrine, paracrine, or autocrine factors converge in a complex stage-specific multifactorial control of spermatogenesis [10, 18–24].

Spermiogenesis is the terminal differentiation process of male germ cells, during which haploid round SPT undergo extensive biochemical and morphological changes including acrosome formation, flagellar development, chromatin condensation, and severe nuclear and cellular reorganization [25, 26]. In mouse, morphological criteria have been used to classify spermiogenesis in 16 developmental steps [27]; in particular, round (steps 5–8), elongating (steps 9–11), condensing (steps 12–14), and condensed (steps 14–16) SPT are differentially characterized by acrosomal and flagellar development, as well as by cellular and nuclear shape. During the early steps, round SPT are transcriptionally active; in elongating SPT, transcriptional activity decreases and then turns off; later, in condensing SPT, an extensive chromatin reorganization occurs at molecular and morphological levels [28].

The underlying events that lead to this extensive chromatin reorganization and packaging have been reported in several excellent reviews and here summarized, but very little is known about the molecular mechanisms involved [26, 29–32]. Interestingly, in recent overviews about estrogens (E_2_) and spermatogenesis in mammals, the presence of E_2_ receptor (ER) and aromatase in somatic and germ cells has been underlined to suggest a possible involvement of this traditionally female hormone in spermiogenesis [2, 33–36]. 

In this review, we focus on the recent advances in our laboratory about the emerging role of E_2_ in SPT chromatin reorganization during spermiogenesis. In particular, new insight has come out from the study of type 1 cannabinoid receptor knockout (Cnr1^−/−^) mice.

## 2. Chromatin Reorganization in SPT

Spermatozoa are highly differentiated haploid cells with a particular chromatin organization that results from remodeling events occurring during meiotic and postmeiotic phases of spermatogenesis [26].

Indeed, when cells enter the meiotic prophase, all the somatic histones, except H4 (i.e., H4t-gene protein), are replaced by testis-specific (TH2A, TH2B, TH3, and H1t) or testis-enriched (H2AX, H1a) histone variants [37–39]. Testicular variants of H1 linker are H1t, H1t2, and HILS1 [40–42]. Among these, H1t has been reported to exert the lowest condensing effect on rat testis oligonucleosomes [43]. The high levels of H1t (about 55% of the linker histones) during the pachytene phase until the stage of elongating SPT suggest a role in keeping chromatin in a relatively decondensed state which enables nuclear events. Indeed, during the early steps, round SPT are transcriptionally active and contain H1t-enriched nucleosomal chromatin. In elongated SPT, H1t persists until the transcriptional activity of the genome is still detectable [37]. This histone-variant incorporation step, together with histone posttranslational modifications, such as acetylation, methylation, ubiquitination, and phosphorylation, creates specific chromatin domains characterized by quickly disassembling nucleosomes and by a new “histone code,” both facilitating histone displacement [39, 44, 45].

**Figure 1:**
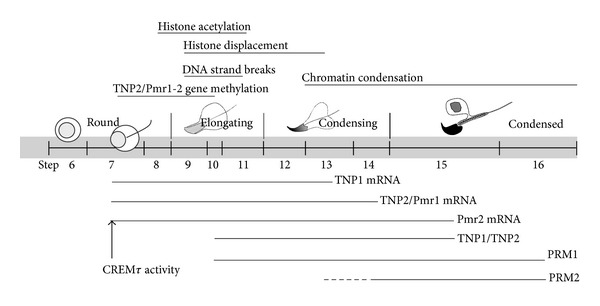
Timing of the main chromatin remodeling events in round, elongating, condensing, and condensed spermatids (the related references are reported in the text).

During the postmeiotic stage of spermatogesis, when round SPT are extensively remodelled to form mature SPZ, a gradual and radical change in the chromatin cytoarchitecture is observed (the main events are summarized in Figure 1) [46]. This extensive chromatin reorganization requires (i) expression and storage of specific proteins involved in condensation, such as transition proteins (TNP) and protamines (PRM), (ii) transient DNA strand-breaks which require the topoisomerase enzyme, (iii) displacement and degradation of the nucleosomal structure, (iv) sequential histone replacement, firstly by TNP and then by PRM, (v) transcriptional silencing and DNA repair, and (vi) repackaging of protaminated chromatin into toroidal structures [47, 48]. However, many species retain a small fraction (1% in mouse, 15% in human) of their chromatin in the more relaxed nucleosomal configuration [49] so that SPZ contain at least two differentially packaged chromatin domains: (1) the PRM-based chromatin that organizes the bulk of DNA in a highly compact toroidal configuration, suitable to arrest transcription and mask genome from exogenous and endogenous damage until fertilization [29]; (2) the nucleosome-based chromatin that organizes epigenetically marked developmental loci in a potentially dynamic transcriptional configuration, useful after fertilization [30, 50]. Interestingly, early after fertilization, before activation of the embryonic genome, the paternal pronucleus becomes highly transcriptionally active compared with the female pronucleus [51].

At molecular level, histone-to-PRM exchange requires the expression and storage of specific mRNA involved in condensation. Indeed, transcription and translation are temporally uncoupled. Tnp1/2 and Pmr1/2 mRNAs are synthesized and stored for some days in SPT and later translated, implying a timely controlled process of haploid-regulated transcription and translation [28, 52, 53]. In particular, Tnp1 and Tnp2 mRNA are preserved in translationally inert ribonucleoprotein particles; afterward, they are translated in elongating-condensing SPT (steps 10–15 of spermiogenesis) and then degraded after translation [52, 53]. Temporal and stage-specific appearance of TNP and PRM is strictly regulated and is prerequisite for the correct differentiation of round SPT into mature and motile SPZ with fertilizing capability [54].

Transcriptional regulation of haploid genes depends on potentiation of gene via association with nuclear matrix attachment regions (MARs) [55]. It also depends on DNA methylation and recruitment of transacting factors like TATA-box protein (TBP), Y-box proteins, and cAMP-responsive element modulator (CREM). The latter is a transcription factor which binds as homo- and heterodimers to the regulatory sequence CRE (cAMP-responsive element) [29, 56]. The CREM-encoding gene is highly expressed in the adult testis and shows multiple site of alternative splicing [57]. Levels of CREM transcripts are low in prepubertal testis and only the repressor isoforms (*α*, *β*) are detected. However, during puberty, transcripts encoding the activator form CREMtau (CREM*τ*) appear abundantly expressed only in germ cells, from the pachytene SPC stage onward, while CREM*τ* protein is not detected in SPC but only in haploid SPT at very high levels [57, 58]. The CREM switch (repressor versus activator) is regulated by the gonadotropin FSH which, acting through Sertoli cells, paracrinally directs the use of an alternative polyadenylation site in SPT, resulting in a more stable CREMt transcript [59]. Many haploid genes have been identified as potential CREM targets since they contain CREs or half CRE in their promoters. Indeed, CREMt regulates gene expression of Tnp and Prm [29, 56].

In mouse, Pmr1, Pmr2, and Tnp2 genes are clustered on chromosome 16 and, contrary to the usual paradigm, they are fully methylated when actively transcribed. In contrast, the Tnp1 gene shows demethylation in the 5′ region associated with gene activity [38]. In humans, the DNA methyltransferase 1 (DNMT1) is restricted to male germ cells (pachytene SPC and round SPT), and infertile patients showing round SPT maturation arrest also show a specific DNMT1 loss in these cells [60]. Similarly, *Crem*-null mice show round SPT maturation arrest [61, 62]. These observations suggest that methylation and CREM are master controllers of SPT differentiation.

In mouse, histone displacement starts at step 9. Main events promoting histone displacement are phosphorylation of histone H1t [63] and hyperacetylation of H4. The latter process has been largely studied in germ cells of several species [64–66] and is largely conserved during evolution. It has been demonstrated that hyperacetylation of histone tails (steps 8–11 SPT) relaxes nucleosomal DNA-histone interaction, and precedes and overlaps either histone displacement or TNP1/TNP2 presence at nuclear level (step 10–early 15 SPT). Accordingly, core-histones are displaced in their acetylated state [66]. Available reports suggest that the process of histone displacement requires (i) DNA nick/repair induced by topoisomerase which relieves torsional stress associated with histone-to-PRM exchange; (ii) hyperacetylation of histone tails by histone acetyl transferase with a concerted down regulation of histone deacetylase; (iii) histone removal mediated by the recruiting protein BRDT (testis-specific bromodomain protein) able to bind histone acetylated lysines, and (iv) acetylated histone degradation through polyubiquitylation of N-terminal lysines [31]. Histone displacement ends at step 13 SPT [47]. 

Concurrently to the aforementioned steps, a DNA-binding competition mechanism leads to histone-to-TNP exchange (TNP1-4 in mice, rats, boars, bulls, and men; the best characterized are TNP1 and TNP2) and to final TNP-to-PRM transition (PRM1 in rats; PRM1 and PRM2 in stallions and mice; PRM 4 in humans) [39, 47]. Phosphorylation and dephosphorylation of TNP and PRM trigger their nuclear translocation, their binding to DNA, and eventually chromatin condensation [28, 67]. It has been demonstrated that TNP1 has important DNA-nucleosome core destabilizing properties because it decreases the melting temperature of DNA and relaxes DNA in nucleosomal core particles *in vitro* [68]. In contrast, TNP2 seems to be a DNA-condensing protein [69] and its phosphorylation by protein kinase A greatly reduces its condensation property [70]. Although TNP1 and TNP2 apparently show distinct functions, together with PRM, both are involved in DNA strand-break repair [53, 71–74] and male mice with single *tnp1* or *tnp2* gene deletion demonstrate that TNP1/2 partially complement each other and both affect PRM2 processing before its binding to DNA [75]. Interestingly, in double *Tnp1/Tnp2*-null mice, histone displacement and PRM deposition proceeded relatively normally, chromatin condensation occurs irregularly, and many SPT show DNA breaks, thus demonstrating that although TNP are required for a normal chromatin condensation, they are not essential for the process [76]. 

Protamines are the most basic DNA-condensing proteins. Most likely, they arise from an ancestral histone H1 gene [77], but, differently from histones, they are characterized by arginine (in eutherians, arginine, and cysteine) rather than lysine residues [78]. This biochemical difference explains PRM greater affinity for DNA, due to a higher hydrogen binding potential of arginine over lysine [79]. These proteins may bind to the major and minor groove of DNA or to the DNA surface by interacting electrostatically with phosphate residues. It has been demonstrated that PRM allow chromatin condensation through arginine residues into toroidal structures at testicular level [29], and further through cysteine residues along the epididymal transit, when inter- and intraprotamines disulphide bonds are formed [80]. In concert with thiol oxidations, PRM also undergo tyrosine phosphorylation during *caput*-to-*cauda* transit [81].

At morphological level, when histone-to-PRM transition occurs, an extraordinary event is observed in the nucleus of differentiating germ cells: flocculent densities of chromatin coalesce into a coarsely granulofibrillar chromatin, which gradually extends in a centripetal and rostral-to-caudal direction and becomes dense and homogeneous at the end of spermiogenesis [28]. This chromatin condensation in toroidal structures modifies the shape of the whole nuclear compartment and strongly reduces its size promoting development of the peculiar elongated, small, and hydrodynamic sperm head that supports swimming ability. Indeed, by stacking these toroids, the sperm nucleus achieves a higher efficiency in packaging the paternal genome and therefore in reducing its size to an absolute minimum [30]. The mechanism by which PRM induce the conformational change in chromatin packaging is not well understood, but it is probably related to PRM properties and to enzymes involved in chromatin remodeling [29, 56]. 

Abnormal sperm histone or PRM content can disrupt chromatin organization [82–84]. Indeed, histone retention decreases nucleoprotamine-based chromatin and exposes a more relaxed chromatin to damage [56, 85]. In both humans and animals, abnormal DNA damage is associated with compromised fertility and increased miscarriage rates [56, 76, 86]. Therefore, chromatin quality is an objective marker of sperm function that provides a significant prognostic factor for male infertility [87–89]. 

## 3. Estrogens and Spermiogenesis

The presence of intracellular (ER*α* and ER*β*) and transmembrane (GPR30) E_2_ receptors in the testis and in particular the expression of ER*β*, GPR30, and aromatase in germ cells have highlighted the physiological role of the E_2_ in spermatogenesis [35, 90]. Aromatase knockouts (ArKO) or ER*α* knockouts (*α*ERKO) have further accentuated the role of E_2_ in germ cell progression and maturation. Indeed, the specific phenotypes have demonstrated that in *α*ERKO mice disruption of spermatogenesis appears to be primarily linked to mechanical defect in the epididymis [1], whereas in ArKO mice a specific depletion in developing SPT seems to occur [91]. To date, no helpful information came from ER*β* or GPR30 knockout mice [2, 33, 34].

Traditionally, testosterone and E_2_ were considered male only and female only hormones, respectively. However, at the beginning of the 1930s, the developmental exposure to high doses of E_2_ was reported to induce malformation of the male reproductive tract in mammals [92], thus suggesting that E_2_ might regulate male reproduction [2, 93]. It is now accepted that E_2_ regulate spermatogenesis (gonocyte and spermatogonia proliferation, meiosis, Sertoli cell function) as well as spermiation, sperm transport, and epididymal sperm maturation. Some of these functions are evolutionarily conserved since they have been observed in mammalian and nonmammalian species [1, 7, 8, 12, 14, 19, 36, 94–99]. 

The first evidence that E_2_ affect spermiogenesis came in 1987, when adult male rats were treated with an ovarian protein able to inhibit aromatase activity. After treatment, animals showed degeneration of round SPT and a massive decrease of elongated SPT [100, 101]. Accordingly, a significant decrease in round and elongated SPT, but not in earlier germ cells, was found in adult male bonnet monkeys treated with an aromatase inhibitor for 150 days [102]. These data are not surprising, given the more intense immunostaining and higher aromatase activity in SPT than in any other testicular cells [103, 104]. 

To well define the role of E_2_ in male germ cell development, mice with a targeted disruption of the *cyp19A1* aromatase gene (ArKO mice) were generated [91]. These animals were initially fertile but developed progressive infertility between 4.5 months and 1 year. Spermatogenesis is primarily arrested at early spermiogenic stages, with the appearance of multinucleated cell into the tubular lumen. Furthermore, an abnormal acrosome development with multiple acrosomal vesicles and uneven spreading over the nuclear surface is also observed [91]. This observation suggests that acrosome biogenesis may be an E_2_-dependent process. Accordingly, aromatase is detected at high levels in the Golgi complex of developing SPT [34]. The progressive nature of the phenotype may be intrinsic to the mechanism of E_2_ action in adult seminiferous epithelium, as observed also in female ArKO mice, characterized by a progressive phenotype too [91]. Alternatively, the delayed phenotype may be explained by the high content of phytoestrogens in the diet, which may supply sufficient exogenous E_2_ to maintain normal spermatogenesis in young animals. Indeed, in young ArKO mice on a phytoestrogens-free diet, the phenotype was more severe than in mice on normal diet [105, 106]. 

However, ArKO mice are not an ideal model to study E_2_-regulated events during spermatogenesis in adulthood because of developmental absence of E_2_. Therefore, using rat and mouse, several attempts were carried out to create conditions of high intratesticular E_2_ levels and study its effect on spermatogenesis.

Earlier studies on long-term exposure to pharmacological doses of E_2_ in adult male rats demonstrated that this treatment suppressed both gonadotropins and testosterone releases and induced complete azoospermia [107]. Then, a second attempt was based on the administration of different doses of exogenous E_2_ over a period of 10 days to increase intratesticular E_2_ levels with a concomitant deficiency in circulating FSH and both plasma and intratesticular testosterone. By exploiting these experimental conditions, it was suggested that, during spermiogenesis, round SPT differentiation (steps 1 to 6) was largely dependent on E_2_, whereas SPT elongation (steps 8 to 19) was androgen dependent [108]. These data were supported by the observation that high intratesticular E_2_ levels preserved round SPT steps 1–6 whereas testosterone deficiency, induced by E_2_ treatment via a negative central feedback, in turn originated pyknotic bodies in elongated/condensed SPT steps 8–19 [108]. In a further study, a similar treatment in rats significantly decreased testicular levels of CREM*τ* protein, as well as the CREM*τ*-inducible TNP1/2 and PRM1 proteins, while the relative mRNA levels were not changed [109]. In the same article, the E_2_ treatment was also reported to significantly increase testicular androgen binding protein (ABP) mRNA levels, thus suggesting a specific stimulatory effect of E_2_ on ABP gene regulation or RNA stability. Authors concluded that E_2_ suppressed the appropriate translation of the spermatidal proteins through an ABP-dependent posttranscriptional mechanism. Surprisingly, no information about testicular morphology was reported [109]. A further confirmation of E_2_ activity on SPT came from the observation that GPR30 regulates expression of apoptotic markers in rat pachytene SPC and round SPT [90, 110]. More interestingly, growing pieces of evidence reveal that endocrine disruptors, that is, environmental pollutants able to interfere with endogenous endocrine system, have been demonstrated to negatively affect spermatogenesis; among these, there are disruptors with agonistic and antagonistic estrogenic effects. To date, this is a matter of debate and concern because these compounds may have damaging outcome on animal and human fertility [111].

Recent findings reveal that E_2_ restore spermatogenesis in hypogonadal (hpg) mice. Due to a natural *Gnrh* gene deletion, the *hpg* mice are functionally deficient in gonadotropins and sex steroids and show meiosis arrest at pachytene stage. Treatment with E_2_ or ER*α* agonist restores meiosis in these animals which, in absence of testosterone, produce haploid elongated SPT, likely via a mechanism involving a weak neuroendocrine activation of FSH secretion [112–114]. Qualitatively complete spermatogenesis could be also restored in *hpg* mice by administration of either testosterone or its metabolite, the potent nonaromatizable androgen dihydrotestosterone (DHT), in absence of FSH stimulation [115]. However, in this case, a possible E_2_ involvement cannot be excluded. Indeed, it has been reported that DHT can be converted in 3*β*-diol by 3*β*-hydroxysteroid dehydrogenase (3*β*-HSD), which preferentially binds ER rather than androgen receptor [116]. Interestingly, in addition to th results obtained in *hpg* mice [112–114], studies on FSH-receptor knockout mice (FORKO) support an E_2_ involvement in spermiogenesis, likely in a synergistic or independent way with FSH and/or androgens [117]. Indeed, FORKO mice show low testosterone and E_2_ levels [118], as well as a significant increase of spermatogonia, decrease of elongated SPT, and weight loss of testis, epididymis, and seminal vesicles. Tubular and luminal diameters of *caput* epididymis appear smaller than those of wild-type males with few lumina filled with SPZ [117]. At molecular level, deprivation of FSH signaling greatly decreases TNP/PRM levels as well as chromatin quality of SPZ [119]. 

We recently characterized the reproductive phenotype of type 1 cannabinoid receptor knockout (*Cnr*1^−/−^) male mice. These mutants exhibit endocrine and phenotypic features which are useful to extend the above studies about the role of E_2_ in SPT differentiation and in particular in the maintenance of sperm chromatin quality. Main features are summarized in Table 1 and described below.

## 4. Reproductive Phenotype of *Cnr1* ^**−/**−^ Male Mice: E_2_ Activity on Sperm Quality

Endocannabinoids are lipidic mediators identified in several peripheral tissues (brain, testis, and epididymis) and biological fluids (follicular fluid, maternal milk, and blood) [4, 124–129]. To date, the best characterized are arachidonoylethanolamide (AEA or anandamide) and 2-arachidonoyl-glycerol (2-AG), but other molecules have been proposed as possible cannabinoid receptor (CNR) agonists [26, 130]. Endocannabinoids regulate reproduction, in both males [16, 17, 20, 26, 122, 131–139] and females [140–145], and specific G-protein-coupled cannabinoid receptors, CNR1 and CNR2 (or CB1 and CB2, resp.), have been localized in male and female reproductive tracts [20, 146–148]. In the testis, CNR1 is present in somatic and germ cells including SPT, from round stage onward [122, 129, 131, 149–154], and recently its involvement in chromatin packaging during SPT differentiation has been reported [26, 85]. However, much remains to be clarified about a direct and/or indirect role. *In vivo* studies, using nonmammalian and mammalian animal models, show that CNR1 acts at both central and local level [155, 156]. In frog, an intriguing CNR1-GnRH (gonadotropin-releasing hormone) interplay occurs at a central level, with CNR1 regulating GnRH synthesis [16]; at testicular level, CNR1 also regulates *GnRH1/2* and *GnRH-R (GnRH-receptor) *expression [157]. In rat, CNR1 regulates the release of hypothalamic GnRH [158], while in mice it increases *Tnp2* expression in the testis [85]. It has been hypothesized [26] that testicular AEA, responsive to E2 and produced by somatic cells [131, 159] and/or by SPT [140], may act as paracrine/autocrine factor on SPT themselves via CNR1, by regulating *Tnp2* mRNA transcription or stability.

Most information about CNR1 involvement in male reproduction came from *Cnr*1^−/−^ mice. An early study reported that *Cnr*1^−/−^ male efficiently synthesizes the gonadotropin LH but shows low levels of LH and testosterone in the bloodstream. Furthermore, *Cnr*1^−/−^ testis produces few testosterone *in vitro* [120]. Recently, we have characterized the reproductive phenotype of *Cnr*1^−/−^ mice and reported that males show normal progression of spermatogenesis [3, 4, 122], produce SPZ [3, 4], and are fertile [85] although they displayed a lot of abnormalities (see Table 1 and references herein) such as (i) downregulation of neuroendocrine axis [121], (ii) developmental decrease of Leydig cell number [122], (iii) low sperm chromatin quality [85, 121], and (iv) abnormal epididymal sperm motility (i.e., potential to move) acquisition [3, 4]. Some of these abnormalities well fit with the early data reported by Wenger et al. [120]. Indeed, at molecular level *Cnr1* gene deletion originates a ligand-dependent downregulation of GnRH-R signaling and this may explain the LH drop originally observed in these animals. In any case, although both LH and testosterone decrease by 50% in serum of *Cnr*1^−/−^ as compared with wild-type mice [120], we found that LH signaling is sufficient to regulate steroidogenesis supporting testosterone production in Leydig cells. Indeed, the 3*β*-HSD, a LH-responsive selective marker of Leydig cells, is synthesized at normal levels in individual single cells [121], thus suggesting that in *Cnr*1^−/−^ mice the testosterone decrease, in both *in vivo* and *in vitro* systems [120], is exclusively related to a decrease of Leydig cell number. We also found that GnRH downregulation is accompanied by downregulation of *Fshb*, *Fsh-R*, *Tnp2*, and *P450arom* mRNA as well as of TNP2 and P450arom protein. The P450arom protein decrease is observed in the interstitial Leydig cells and the low levels are independent by Leydig cells number. Simultaneously, low E_2_ levels were detected in the bloodstream suggesting that, in the mutant mice, the downregulation of neuroendocrine axis interferes with gene expression of Tnp2 in SPT as well as of P450arom in Leydig rather than in germ cells with consequent reduction of E_2_ levels in the bloodstream [121]. 

The morphological and biochemical evaluations of epididymal *Cnr*1^−/−^ sperm samples showed a lot of abnormalities. In wild-type animals, a 2-AG gradient (high level in *caput *versus low levels in* cauda*) prevents sperm motility acquisition in *caput* through the inhibitory activation of CNR1. In knockout animals, a high number of SPZ from *caput* epididymis appears motile, thus demonstrating that, in absence of CNR1, SPZ precociously acquire their potential to move [4]. The morphological and biochemical analyses of epididymal *Cnr*1^−/−^ SPZ also showed poor chromatin quality [26, 85]. In particular, we found that the genetic inactivation of *Cnr1* affects chromatin remodeling mechanisms that occur in SPT during spermiogenesis. Indeed, in *caput* epididymis from *Cnr*1^−/−^ animals, the number of SPZ with histone retention as well as the number of SPZ with uncondensed chromatin or with DNA damage is higher than in *Cnr*1^+/+^ and *Cnr*1^+/−^ animals [85, 121, 123] demonstrating that in these animals spermiogenesis is qualitatively inefficient. Despite that, animals retain their fertility [85], likely because of a sufficient number of SPZ with mature chromatin. Correlation analysis and morphological studies also showed that abnormal histone retention is strictly related to uncondensed chromatin or DNA damage as well as it is associated to sperm nuclear size elongation. Intriguingly, histone displacement and chromatin condensation normally occur in a rostral-to-caudal direction [89] and then the failure of these mechanisms might be responsible of the nuclear swelling along its longitudinal axes. Recent experiments suggest that low plasma E_2_ levels might be the cause of the sperm chromatin imperfections observed in these animals [123]. Indeed, 24-day postpartum *Cnr*1^−/−^ male mice exposed to low doses of E_2_, every other day for a complete cycle of spermatogenesis, showed a weak upregulation of neuroendocrine axis (no effect on *GnRH* mRNA levels; strong increase of *GnRH-R* mRNA; weak increase of *Fshb* subunit, *Fsh-R*, and *P450arom* mRNA; weak increase, about 20%, of P450arom protein in Leydig cells; no effect on *Tnp2* mRNA) and the rescue of sperm chromatin quality indices (histone content, chromatin condensation, DNA damage, and nuclear size), via an ER mediated mechanism [121]. Several studies propose that chromatin condensation and DNA damage are related to each other and are secondary effects associated to disrupted histone displacement [29, 56]. Therefore, it is plausible to conclude that E_2_ treatment, through a TNP2-independent effect, primarily affects histone displacement and sequentially induce the rescue of sperm chromatin quality indices to physiological values. In agreement, *caput* SPZ from rat chronically injected with E_2_ showed a TNP/PRM1-independent chromatin hypercompaction [109]. In addition, serum concentration of E_2_ and free T4 inversely correlates with sperm DNA damage in men from an infertility clinic [160]. Furthermore, it has been reported that E_2_ delay testicular cell damage, which leads to functional senescence. Therefore, E_2_ are helpful in protecting the reproductive functions from the adverse effects exerted by reactive oxygen species (ROS) produced in large quantities in the aged testis [161].

These results, in combination with aforementioned data, show that E_2_, indirectly via stimulatory effects on FSH secretion and/or directly via paracrine actions within the testis, play a key role in spermiogenesis since they preserve chromatin packaging in SPT and then sperm quality. Interestingly, sperm nuclear length, which is related to chromatin quality, appears to be an E_2_-responsive morphological parameter [123] and may be used as helpful tool to discriminate “in real time,” among morphologically normal SPZ, those with a good chromatin quality. To date, no tool exists to verify “in real time” sperm chromatin quality. Interestingly, in assisted reproduction technique field, abnormal nuclear size of human SPZ is commonly considered to be of poor prognosis [162]. More interestingly, a recent article describes a tight correlation between percentage of SPZ with nuclear form abnormalities, screened by MSOME (motile sperm organelle morphology examination) technique, and DNA fragmentation [163].

## 5. Last Considerations and Conclusions

Studies on *α*ERKO mice led to conclude that E_2_ are involved in epididymal sperm maturation. Our data and those reported in the literature suggest a further and intriguing role for E_2_ in spermiogenesis and in maintenance of sperm chromatin quality. The main future endpoint will be the characterization of E_2_ mechanisms to better understand whether its action is direct and/or mediated. Indeed, it is still a matter of debate whether E_2_ and/or FSH affect chromatin remodeling in SPT in either a synergistic or an independent way with androgens [31]. Gene deletion animal models have revealed that both FSH and testosterone levels are implicated in the regulation of chromatin condensation during spermiogenesis [31]. However, although is emerging idea is that these hormones may act in synergy [31, 164], it has been reported in rat that the inhibition of FSH, resulted from hyperprolactinemia induction, reduces chromatin packaging in an androgen-independent way.

Apart from the divergences, FORKO mice exhibit endocrine and phenotypic features of *Cnr*1^−/−^ male mice, including reduced histone displacement, enlarged sperm head size, decreased chromatin quality of SPZ (low packaging and high DNA damage), and low levels of testosterone and E_2_ [117–119]. In *Cnr*1^−/−^, the number of SPZ and epididymal epithelium morphology, both dependent on testosterone [165], are not affected [3] suggesting that, in mutant mice, testosterone ranges within levels sufficient to support spermatogenesis. Therefore, we speculate that, in *Cnr*1^−/−^ mice, E_2_ action, (i.e., the rescue of histone displacement in SPT and chromatin quality in SPZ) is independent of testosterone. Our future endpoint will be to confirm if this really occurs and to establish E_2_ and FSH roles. The importance of these studies is corroborated by the growing pieces of evidence that E_2_ activity can be mimicked or antagonized by estrogenic environmental chemicals with potentially damaging effects on animal and human fertility [111].

## Figures and Tables

**Table 1:** Reproductive phenotype of *Cnr*1^−/−^ male mice and related references.

*Hypothalamus-pituitary-testis axis *	
(i) Normal pituitary LH content [120]	
(ii) Low serum LH concentration [120]	
(iii) Low testicular testosterone secretion [120]	
(iv) Low circulating testosterone and E_2_ levels [120, 121]	
(v) High pituitary GnRH-R and low FSH*β* subunit mRNA levels [121]	
(vi) Low testicular Fsh-R mRNA levels [121]	
(vii) Low testicular P450 mRNA levels [121]	
(viii) Low P450arom levels in Leydig cells [121]	
(ix) Low Tnp2 levels (both mRNA and protein) [85]	
*Adult Leydig cell *	
(i) Low number of adult Leydig cells [122]	
(ii) Normal 3-*β*Hsd mRNA levels/Leydig cell [121]	
*Sperm chromatin quality *	
(i) High number of SPZ with retained histones [85, 121, 123]	
(ii) High number of SPZ with uncondensed chromatin [85, 123]	
(iii) High number of SPZ with DNA damage [85, 123]	
(iv) SPZ with high % of damaged DNA [85]	
(v) Increase of DNA damage during epididymal transit from *caput* to *cauda* [85]	
(vi) High mean values of sperm nuclear length [123]	
*Epididymal sperm motility acquisition *	
(i) High number of potentially motile SPZ in *caput* [4]	
(ii) Precocious sperm motility acquisition in *caput* epididymis [4]	

## Abbreviations

SPC: Spermatocytes
SPT: Spermatids
SPZ: Spermatozoa
E_2_: Estrogens
ER: Estrogen receptor
Cnr1^−/−^: Type 1 cannabinoid receptor knockout
TNP: Transition proteins
PRM: Protamines
MARs: Nuclear matrix attachment regions
TBP: TATA-box protein
CREM: cAMP-responsive element modulator
CRE: cAMP-responsive element
CREM*τ*: CREMtau
DNMT1: DNA methyltransferase 1
BRDT: Testis-specific bromodomain protein
GPR30: Transmembrane E2 receptor
ArKO: Aromatase knockouts
*α*ERKO: ER*α* knockouts
ABP: Androgen binding protein
Hpg: Hypogonadal
DHT: Dihydrotestosterone
3*β*-HSD: 3*β*-Hydroxysteroid dehydrogenase
AEA: Arachidonoylethanolamide
2-AG: 2-Arachidonoyl-glycerol
CNR: Cannabinoid receptor
GNRH: Gonadotropin-releasing hormone
GNRH-R: GnRH-receptor
ROS: Reactive oxygen species
MSOME: Motile sperm organelle morphology examination
FORKO: FSH-receptor knockout mice.
